# Mental heartbeat tracking and rating of emotional pictures are not related

**DOI:** 10.1007/s00426-021-01593-4

**Published:** 2021-09-23

**Authors:** Eszter Ferentzi, Luca Vig, Mats Julin Lindkjølen, Markus Erling Lien, Ferenc Köteles

**Affiliations:** 1 Doctoral School of Psychology, ELTE Eötvös Loránd University, Budapest, Hungary; 2 Institute of Health Promotion and Sport Sciences, ELTE Eötvös Loránd University, Budapest, Hungary; 3 Károli Gáspár University of the Reformed Church in Hungary, Budapest, Hungary; 4 Institute of Psychology, ELTE Eötvös Loránd University, Budapest, Hungary

## Abstract

Our aim was to conceptually replicate the findings of previous empirical studies showing that people with higher cardiac interoceptive accuracy experience more intense emotions. Apart of the mental heartbeat tracking task of Schandry, Hungarian (*n* = 46, 76.0% female, mean age 22.28 ± 2.228) and Norwegian (*n* = 50, 60.0% female, mean age 24.66 ± 3.048) participants rated the arousal and valence evoked by positive, neutral and negative pictures. Multivariate repeated analysis of variance (applying both frequentist and Bayesian approaches) did not reveal any connection between heartbeat perception scores and the subjective ratings (i.e., arousal and valence) of the pictures in any of the two groups. The lack of the expected association between cardioceptive accuracy and arousal might partly be explained by the methodological differences between previous studies and this one; for example, we did not split or preselected the sample based on the performance on the Schandry task and applied a relatively strict instruction (i.e., by encouraging to count felt heartbeats only, and to report zero if no sensations were detected).

**Supplementary Information:**

The online version contains supplementary material available at 10.1007/s00426-021-01593-4.

## Introduction

Interoception is described both as the representation of the physiological feedback from the whole body, and its perception (Wiens, 2005). It can be assessed by sensory tasks, for example by heartbeat perception paradigms (also called interoceptive accuracy, IAc), and by questionnaires (called interoceptive sensibility, ISb) (Garfinkel et al., 2015).

Interoception is supposed to substantially contribute to affective processes, primarily to subjective experience (Wiens, 2005). According to the so called circumplex model of affect, affective experience consists of two major and more or less independent aspects, arousal and valence (Barrett & Russell, 1999; Kuppens et al., 2013; Russell, 1980; Watson et al., 1999). Empirical results indicate that people who perceive their heartbeat better report higher arousal when they rate film clips (Wiens et al., 2000) and pictures (Herbert et al., 2007, 2010; Pollatos et al., 2005, 2007a), as compared to poor heartbeat perceivers. However, no differences between good and poor perceivers were found with respect to valence. In other words, cardiac IAc appears to contribute to the perceived intensity of emotional experience, regardless of the valance of the emotion at hand.

Although the findings seem decisive at first sight, several reasons call for replication. First, the majority of the studies is not without methodological limitations; this makes the conclusion less clear-cut. Among the studies that investigated this topic, only the minority (e.g., Wiens et al., 2000) applied the heartbeat detection task, an assessment of IAc that is based on a force choice paradigm (Eichler & Katkin, 1994; Whitehead et al., 1977). The vast majority of the studies used the mental heartbeat tracking task of Schandry (1981). The debate about the validity of the heartbeat perception tasks if far from reaching a conclusion, both the Schandry task (see, e.g., Ainley et al., 2020; Corneille et al., 2020; Flynn & Clemens, 1988; Montgomery & Jones, 1984; Ring & Brener, 1996; Zamariola et al., 2018; Zimprich et al., 2020) and the discrimination tasks (see, e.g., Carroll, 1977; Couto et al., 2015; Pennebaker & Hoover, 1984) have been criticised extensively. It is also worth to mention that a recent meta-analysis questions the interchangeability of the two paradigms (i.e., tracking and discrimination, see, Hickman et al., 2020). In the following, we are going to focus only on studies that apply the mental tracking task, as these represent the majority of the related studies.

Among these, a number of studies worked with relatively low total sample sizes (n ranged between 37 and 44, typically split into two groups) (Herbert et al., 2007, 2010; Pollatos et al., 2005, 2007a), or assessed neutral and unpleasant pictures only (Pollatos et al., 2007b). In addition, a common practice among these studies is to compare people with different level of cardiac IAc (i.e., good vs. poor heartbeat perceivers) (Herbert et al., 2007, 2010; Pollatos et al., 2005, 2007a), either applying preselection or by splitting the sample based on their performance during the heartbeat perception task. This practice, even if it is reasonable under certain circumstances (i.e., when the sample is relatively small), might be misleading because of the limited generalizability of the findings and the loss of information due to splitting.

Second, some other studies contradict the finding that cardiac IAc contributes to the intensity of emotional experience. Research with a focus on affect intensity shows that self-rated intensity is not necessarily related to the physiological arousal (Blascovich et al., 1992; Colombetti & Harrison, 2018). In addition, people seem to differ in their tendency to pay attention to the positive–negative (or hedonic) and to the arousal components of their affective experience, called valence focus and arousal focus (Feldman, 1995); thus, they do not necessarily perceive their bodily processes differently, but interpret them differently. This line of research investigates affect intensity from the viewpoint of social psychology, but some rare examples involve measures of interoception: Blascovich et al. (1992) even found that affect intensity was negatively related to cardiac IAc (assessed with heartbeat detection task).

Thirdly, and finally, although some studies found that the formulation of the instruction of the Schandry task does influence the cardiac IAc scores (Desmedt et al., 2018; Ehlers et al., 1995), it is not common to report its exact wording. Reported instructions of the Schandry task differ to a great extent. In recent studies, the emphasis is on the reduction of non-interoceptive factors (see below), thus the instruction explicitly prohibits estimation and encourages participants to count sensed heartbeats only (Desmedt et al., 2020). This can also lead to biases (i.e., ignorance of weak sensations), thus a more balanced instruction that prohibits estimation but at the same time emphasizes the importance of counting weak sensations is also used (see, e.g., Ferentzi et al., 2018a). In other studies, estimation is allowed if there is no heartbeat-related sensation (Ferentzi et al., 2021), similarly to one of the studies that investigated the Schandry task and the rating of the IAPS pictures (Pollatos et al., 2007a), encouraging participants to „try to count in synchrony with their heartbeats” if “sensory perception of heart activity was lacking” (p. 119). It is worth to mention, that the original study (Schandry, 1981) also allowed estimation, probably to improve the sensation of near-threshold stimuli. This method, however, might also strengthen the influence of the biasing factors (Desmedt et al., 2018; Ehlers et al., 1995); therefore, we support the usage of an instruction that encourage the counting of slight sensations, but also to report zero.

In the present study, our aim was to conceptually replicate previous findings concerning the associations between cardiac IAc (as assessed by the mental tracking task of Schandry, 1981) and perceived components of affective responses (i.e., valence and arousal). To achieve this goal, two independent samples were recruited, no preselection took place and the Schandry task was administered with a relatively strict but balanced instruction (i.e., participants were encouraged to count felt heartbeats only, and to report zero if they do not feel any heartbeats). It was hypothesized that even with this methodological modifications, cardiac IAc would show a moderate positive association with arousal ratings but not with valence ratings.

## Materials and methods

### Participants

The average of correlations (effect sizes) between arousal and the Schandry score reported in previous studies is 0.44 (Herbert et al., 2007, 2010; Pollatos et al., 2005, 2007a, 2007b). A priori sample size calculation for medium level correlation (*r* = 0.44; *α* = 0.05; 1−*β* = 0.90, one-tailed) indicated a minimum required sample size of 41, using the G*Power v3.1.9.2. software (Faul et al., 2007). Overall, 96 undergraduate and graduate university students belonging to a Hungarian and a Norwegian group participated in the study (Hungarians: *n* = 46, 76.0% female, mean age 22.28 ± 2.228: Norwegians: *n* = 50, 60.0% female, mean age 24.66 ± 3.048). Hungarian participants completed the study in Hungarian, while Norwegians did so in English; the latter sub-sample was enrolled from English-speaking university programs in Hungary. Participation was voluntary and anonymous; participants signed an informed consent form. Exclusion criteria were self-reported mental-disorder and being under the influence of alcohol or any medication (except for contraceptives) during the measurement. The study was approved by the ethical committee of the university.

### Procedure

The assessment started with the Schandry task and continued with the presentation of the pictures.

### Heartbeat perception task

Cardiac IAc was assessed with the modified version of the Schandry task (Schandry, 1981). Participants had to silently count their felt heartbeats during three time period (30, 45 and 60 s in random order), while sitting in a chair in a comfortable position, with both legs on the ground. The task started with a 15 s long introductory phase, and there was a 15 s long resting period between each trial. Participants were instructed to count only those heartbeats that were felt. They were encouraged to report zero if they did not have any sensations and to count the heartbeats in case of slight sensations (for the exact instruction, see the Supplementary material). Out of 96 participants, 9 (9.4%) reported zero value at least ones (30 s long interval–8 participants, 45 s long interval–8 participants, 60 s long interval–5 participants). The following formula was calculated for each interval: 1 − [|recorded heartbeats − counted heartbeats|)/recorded heartbeats], and the mean score of the three results provided the cardiac IAc for each participants, ranging from 0 to 1. Higher scores represent higher level of cardioception. Cronbach’s alpha coefficient was 0.848 for Norwegians and 0.944 for Hungarians.

Actual heartbeats were recorded with the Polar WearLink transmitter (RS400) for the majority of the participants (*n* = 81), and due to technical reasons, with the NeXus recording system for the rest (NeXus Wireless Physiological Monitoring and Feedback: NeXus-10 Mark II, Version 1.02; BioTrace + Software for NeXus-10 Version: V201581; Mind Media BV, Herten, the Netherlands). As heartbeats are robust signals and both devices are able to reliably detect individual heartbeats based on the electrical activity of the heart, we did not expect any impact on the results due to the difference of the applied recording systems.

### Pictures

Emotional valence and arousal were assessed by the presentation of 30 pictures belonging to three conditions (10 positive, 10 neutral and 10 negative, in random order) from the International Affective Picture system (Lang et al., 2008), using the OpenSesame software (ver. 3.1). The pictures used were the following. Negative: No. 2095; 2683; 3022; 3063; 3140; 3350; 6520; 9183; 9253; 9405 and 9571. Neutral: No. 1903; 2102; 2377; 2484; 2487; 5390; 5740; 7235; 7175 and 7185. Positive: No. 1460; 1710; 2050; 2340; 5825; 7330; 8170; 8190; 8420 and 8501. Each picture was presented for 6 s. For descriptive statistics of the mean arousal and valence ratings of the pictures (per valence groups and per nationalities), see Table 1. After presentation, each picture was rated both on emotional valence and emotional arousal on a Likert scale from 1 to 9. The task started with 3 introductory pictures (No. 5900; 7595; 8465).

**Table 1:** Descriptive statistics of the assessed variables and zero-order correlations between the Schandry score and arousal and valence ratings in the two groups

	Mean ± Std. Deviation	Correlation with Schandry scores (Spearman’s rho coefficients; *p*)
Hungarians (*n* = 46)		
Schandry score	0.492 ± 0.401	–
Average positive arousal	4.904 ± 1.248	− 0.109; 0.469
Average neutral arousal	3.354 ± 0.831	− 0.013; 0.933
Average negative arousal	7.802 ± 0.728	− 0.159; 0.291
Average positive valence	7.309 ± 0.655	− 0.197; 0.190
Average neutral valence	5.700 ± 0.532	− 0.094; 0.536
Average negative valence	1.589 ± 0.444	0.180; 0.232
Norwegians (*n* = 50)		
Schandry score	0.590 ± 0.368	–
Average positive arousal	4.946 ± 0.938	0.085; 0.558
Average neutral arousal	4.004 ± 0.910	0.000; 1.000
Average negative arousal	6.890 ± 0.909	− 0.024; 0.871
Average positive valence	7.234 ± 0.709	− 0.108; 0.455
Average neutral valence	4.456 ± 0.524	− 0.008; 0.955
Average negative valence	2.400 ± 0.737	0.102; 0.479

### Statistical analysis

Statistical analysis was carried out using the JASP v0.14 software (JASP Team, 2020). Nationality and sex-related differences in the Schandry score were estimated with Student’s *t* tests. Effects of the experimental manipulation were checked with separate repeated-measures analyses of variance (ANOVA) for arousal and valence ratings in both groups. Finally, the hypothesized associations between cardiac IAc and arousal/valence were investigated with another repeated measures ANOVA, with condition (positive, neutral, negative) and aspect (arousal, valence) as within-subject factors, nationality as between-subject factor, and cardiac IAc as covariant. This last ANOVA was conducted with both the frequentist and Bayesian approaches. For these analyses, cardiac IAc was transformed to better fit normality (demeaned values were divided by the Gaussian membership values of the same demeaned values, and the effect of the demeaning was reset by adding the mean of the original data). In the frequentist ANOVA, the Greenhouse–Geisser sphericity correction was applied. In the Bayesian approach, the null model included nationality, arousal and valence ratings, and the interactions terms of these variables, whereas the alternative model included cardiac IAc only. Results are presented as Bayes factors (BF_10_), showing the likelihood of the alternative hypothesis as compared to the null hypothesis. BF_10_ between 1 and 3 represent anecdotal, 3–10 substantial, 10–30 strong, 30–100 very strong and > 100 decisive evidence in favour of the alternative hypothesis (Jarosz & Wiley, 2014).

## Results

**Fig. 1:**
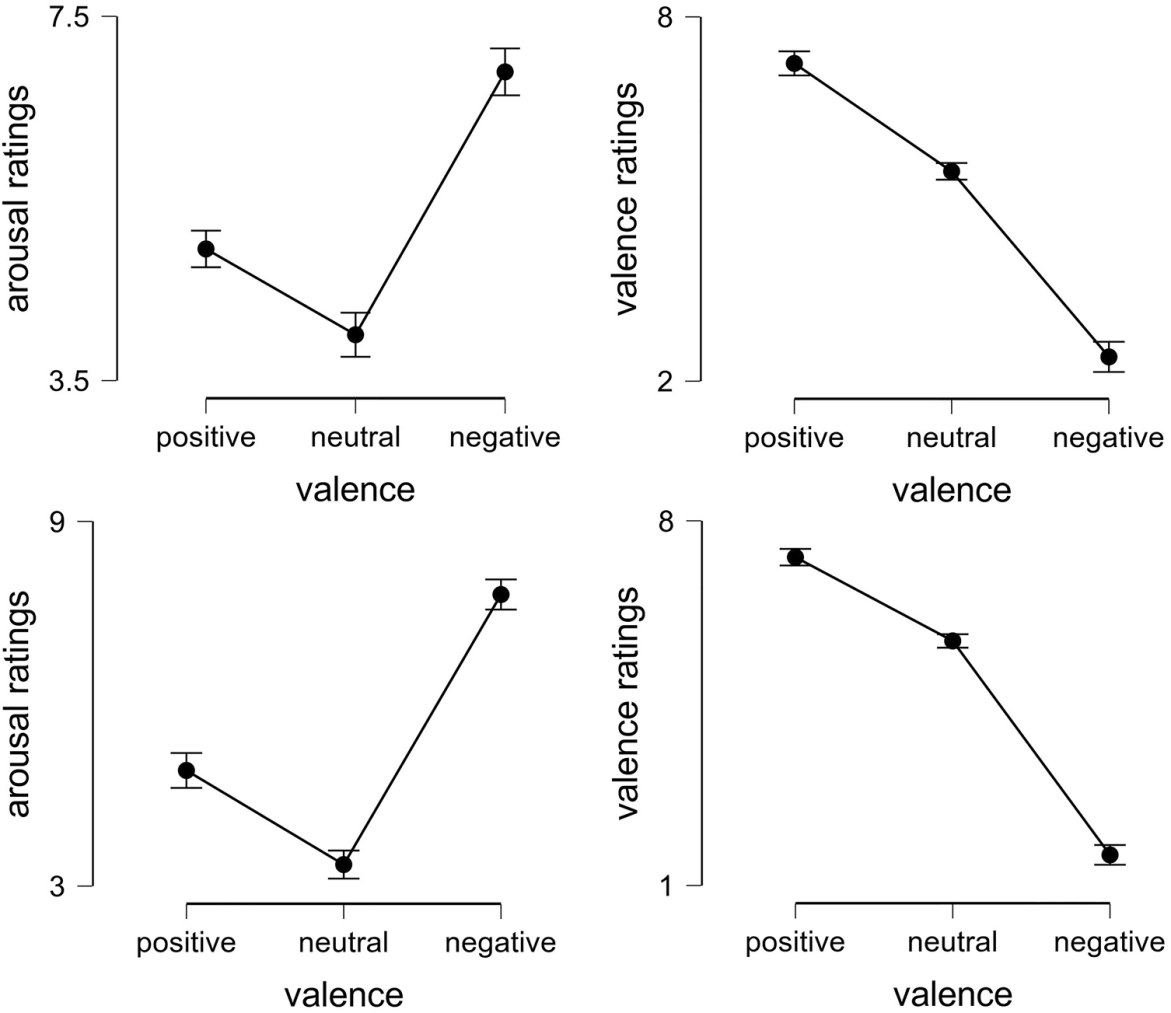
Arousal and valence ratings of the two groups in the three conditions (upper row: Norwegians, lower row: Hungarians). Error bars indicate 95% confidence intervals

Table 1 shows the descriptive statistics of the Schandry score, and arousal and valence ratings of the pictures split by group. No significant difference between the two nationalities with respect to cardiac IAc was found (*t*(94) =  − 1.246; *p* = 0.216, *d* = − 0.255). Similarly, no sex differences were revealed in the total sample with respect to cardiac IAc (*t*(94) = 0.318; *p* = 0.751, *d* = 0.069). Repeated-measures ANOVAs revealed significant differences with respect to arousal ratings in the three conditions for Norwegians (*F*(2,98) = 159.016; *p* < 0.001; *η*^2^ = 0.635) and Hungarians (*F*(2,90) = 313.650; *p* < 0.001; *η*^2^ = 0.790). Similarly, significant differences among the conditions in the valence ratings for Norwegians (*F*(2,98) = 604.275; *p* < 0.001; *η*^2^ = 0.902) and Hungarians (*F*(2,90) = 1351.138; *p* < 0.001; *η*^2^ = 0.951) were revealed (see Fig. 1). Bonferroni corrected post hoc analysis indicated significant (*p* < 0.05) differences among conditions for all cases. Overall, these results indicate that the affective stimuli evoked the expected changes in both groups, similarly to previous studies (e.g., Herbert et al., 2007; Pollatos et al., 2005).

The frequentist repeated measures ANOVA handling all variables together showed a significant condition (positive vs. neutral vs. negative stimuli) main effect (*F*(1.794,166.888) = 137.621; *p* < 0.001; *η*^2^ = 0.093) and an aspect (valence vs. arousal) main effect (*F*(1,93) = 11.409; *p* = 0.001; *η*^2^ = 0.008). These findings are in accordance with the results of individual ANOVAs presented above. Concerning between-subject factors, neither nationality (*F*(1,93) = 0.425; *p* = 0.516; *η*^2^ = 0.005) nor the Schandry score (*F*(1,93) = 0.070; *p* = 0.792; *η*^2^ = 0.001) was significant. Furthermore, no significant interaction between the Schandry score and condition (*F*(1.794,166.888) = 1.708; *p* = 0.187; *η*^2^ = 0.001), aspect (*F*(1,93) = 0.448; *p* = 0.505; *η*^2^ = 0.000), and condition and aspect (*F*(1.663,154.674) = 0.749; *p* = 0.452; *η*^2^ = 0.001) was found.

In the Bayesian approach, repeated measures ANOVA yielded a Bayes factor of 0.139, indicating that the null model is substantially more likely than the alternative model; in other words, the model including IAc proved to be inferior to the null model.

## Discussion

In two independent samples of young individuals, our results show that cardiac IAc, as assessed by the Schandry task with the use of a comparatively strict but balanced instruction, does not relate either to subjective arousal or valance ratings of emotion evoking pictures.

These findings concerning arousal ratings contradict previous results. A study from Pollatos et al. (2007a) showed that cardiac IAc was moderately associated with the mean arousal rating of negative and positive pictures. Another study found an association of similar magnitude between heartbeat perception score and mean arousal score (Pollatos et al., 2005), while two other ones reported moderate to strong relations between cardiac IAc and perceived arousal for positive and negative pictures (Herbert et al., 2007, 2010). All of these studies included cardiac IAc as a binary variable, i.e., as groups of poor and good perceivers, applying a theoretically established threshold criterion, i.e., a Schandry score higher than 0.85 for good perceivers. It is important to see that this criterion is not applicable for scores obtained with a stricter instruction, whereas the mean Schandry score is about 0.7 in these studies, it is about 0.5 in studies with a relatively strict but balanced criterion (Ferentzi et al., 2018a, 2018b), including the present study. Taking these points into consideration, the present study applied a correlational approach which handles IAc as a continuous variable. Another explanation for the lack of association between cardiac IAc and arousal is that the association is substantially weaker than assumed in the a priori sample size analysis. However, the Bayesian analysis applied in our study also supported the lack of association. It is important to note that another previous study with a large samples size (*n* = 102) and using the correlational approach, reported a strong association (*r* = 0.5, *p* < 0.001) between heartbeat perception and mean arousal scores only for unpleasant pictures (Pollatos et al., 2007b).

Our results concerning valence ratings, however, were in accordance with previous findings that could not reveal a significant connection between the valence rating of positive (Herbert et al., 2007, 2010) and negative pictures (Herbert et al., 2007, 2010; Pollatos et al., 2007b) and heartbeat perception score of the mental tracking task. Again, the Bayesian analysis applied in the present study indicates that the lack of such a connection is the most probable state of affairs. To reach a final conclusion on this, the repetition of these studies is needed, covering all types of valence ratings with the investigation of larger samples.

The differences between our findings and those reported in the literature may partly rely on the differences in the applied instruction. Findings indicate that the heartbeat tracking task of Schandry overestimates participants’ performance if the instruction does not explicitly prohibit the estimation of the number of counted heartbeats (Desmedt et al., 2018; Ehlers & Breuer, 1996). The instruction applied in our study encourages participants to report 0 if they do not perceive their heartbeats. In general, top-down cognitive factors, such as participants’ knowledge or belief on their heart rate, seem substantially contribute to their performance (Ring & Brener, 1996, 2018; Windmann et al., 1999). Thus, any attempt to decrease this influence also heavily influence the validity of results obtained with the Schandry task.

The usual interpretation of the findings of previous studies (Herbert et al., 2007, 2010; Pollatos et al., 2005, 2007a, 2007b; Wiens et al., 2000) is that while arousal rating is associated with cardiac IAc (i.e., the accurate perception of bodily processes, including also the changes), valence is not. This is so partly because the exact nature of the felt change is highly context-dependent, and partly because judging pleasantness might relate to the sensitivity of other interoceptive channels, not heartbeats. In addition, heartbeat represents a comparatively neutral signal (i.e., it is not associated either positive or negative emotions) for healthy individuals (Nummenmaa et al., 2018).

It is well-known, however, that the association between actual (i.e., physiological) and perceived arousal is quite low, particularly if the former is not intense (Colombetti & Harrison, 2018). Perceived arousal can be substantially influenced by top-down processes, e.g., expectations (Köteles & Babulka, 2014) and false feedback on physiological changes (Piccione & Veitch, 1979; Valins, 1966). IAPS pictures (Lang et al., 2008) have a very clear message regarding the type of the emotion, which might evoke high demand characteristics. This might be also the case with the Schandry task; studies that do not explicitly prohibit guessing might provoke higher motive to cooperation in participants (Desmedt et al., 2018).

It is common to refer to early theories on emotions (James, 1884, 1890; Lange, 1885) and to their neojamesian representatives (see: Lang, 1994) when the connection of interoception and emotional experience is investigated. These theories, however, are not very explicit regarding the types of relation that can be expected between physiological and psychological processes, so there is almost no limitation how this tight connection could be operationalised in empirical studies. The mere statement (for example) that some kind of connection between physiological changes and their perception can be expected is too broad for a strong theoretical background, i.e., it is not plausible that a broad theory like this could possibly fail the test (Vanpaemel, 2020). In addition, the Schandry task with an instruction requiring the counting of the certainly perceived heartbeats only might not assess near threshold stimuli. It is an open question whether bodily signals at such high level of consciousness play a significant role in emotional experience. We have, however, a good reason to suspect that near threshold stimuli do also have a significant role. These are better measured with the discrimination paradigm. Interestingly, studies applying this method revealed similar results (see, e.g., Wiens et al., 2000) as the ones with the Schandry task, despite their differences (Hickman et al., 2020). To get a clear picture, the further investigation of the subject including both heartbeat perception paradigms would be worthy.

The main limitation of our study is that without the assessment of various physiological variables we do not have information about the objective bodily changes. In addition, we did not include both the classical and the more restrictive forms of instruction for the Schandry task to make a direct comparison. Additionaly, we used a 9-point-scale to assess the impact of IAPS pictures, whereas previous studies applied the Self-Assessment Manikin (Herbert et al., 2007, 2010; Pollatos et al., 2005, 2007a, 2007b). On the one hand, one might suspect that this difference could affect the results; but on the other hand, according to our results the affective stimuli evoked the expected changes in both groups (see above).

In summary, according to our results, the subjective arousal and valence ratings of the emotion evoking pictures are not related to the level of cardiac IAc as it is assessed by the Schandry task with a relatively strict but balanced instruction. In future studies, the control of more potentially influencing variables would be fruitful, involving a large, heterogeneous sample.

## Supplementary Information

Below is the link to the electronic supplementary material.

Supplementary file1 (DOCX 12 KB)
