# Supplementary material for: Mental heartbeat tracking and rating of emotional pictures are not related
Source: Psychol Res. 2021 Sep 23;86(5):1487–94. doi: 10.1007/s00426-021-01593-4 (PMC9177488; doi:10.1007/s00426-021-01593-4)
Supplement: Supplementary file 1 — Supplementary file1 (DOCX 12 KB) [file 426_2021_1593_MOESM1_ESM.docx]

**Supplementary material – Instruction of the Schandry task**

“Sit comfortably, with both feet on the ground, hands on thighs. I will ask you to count your heartbeats. Start counting when I say NOW; and when I say STOP, tell me, how many heartbeats you counted. It is important that you only report the heartbeats that you really sensed. You can feel your heartbeats in many places, for example in your chest or neck, or where your clothes are tighter. It is absolutely OK, if you do not feel anything; in this case, say zero. If you have a slight sensation, you can count it. During the task, your eyes can be open or closed, whatever suits you better. There will three sessions, after a practice session. Is everything clear?”
